# Characterizing the human intestinal chondroitin sulfate glycosaminoglycan sulfation signature in inflammatory bowel disease

**DOI:** 10.1038/s41598-024-60959-x

**Published:** 2024-05-23

**Authors:** Kendra L. Francis, Hengqi B. Zheng, David L. Suskind, Taylor A. Murphree, Bao Anh Phan, Emily Quah, Aarun S. Hendrickson, Xisheng Zhou, Mason Nuding, Alexandra S. Hudson, Miklos Guttman, Gregory J. Morton, Michael W. Schwartz, Kimberly M. Alonge, Jarrad M. Scarlett

**Affiliations:** 1 Department of Pediatric Gastroenterology and Hepatology, Seattle Children’s Hospital, Seattle, WA USA; 2 Department of Medicine, University of Washington Medicine Diabetes Institute, 750 Republican St, Box 358062, Seattle, WA 98195 USA; 3 Department of Medicinal Chemistry, University of Washington, Seattle, WA USA

**Keywords:** Gastrointestinal diseases, Glycobiology

## Abstract

The intestinal extracellular matrix (ECM) helps maintain appropriate tissue barrier function and regulate host-microbial interactions. Chondroitin sulfate- and dermatan sulfate-glycosaminoglycans (CS/DS-GAGs) are integral components of the intestinal ECM, and alterations in CS/DS-GAGs have been shown to significantly influence biological functions. Although pathologic ECM remodeling is implicated in inflammatory bowel disease (IBD), it is unknown whether changes in the intestinal CS/DS-GAG composition are also linked to IBD in humans. Our aim was to characterize changes in the intestinal ECM CS/DS-GAG composition in intestinal biopsy samples from patients with IBD using mass spectrometry. We characterized intestinal CS/DS-GAGs in 69 pediatric and young adult patients (n = 13 control, n = 32 active IBD, n = 24 IBD in remission) and 6 adult patients. Here, we report that patients with active IBD exhibit a significant decrease in the relative abundance of CS/DS isomers associated with matrix stability (CS-A and DS) compared to controls, while isomers implicated in matrix instability and inflammation (CS-C and CS-E) were significantly increased. This imbalance of intestinal CS/DS isomers was restored among patients in clinical remission. Moreover, the abundance of pro-stabilizing CS/DS isomers negatively correlated with clinical disease activity scores, whereas both pro-inflammatory CS-C and CS-E content positively correlated with disease activity scores. Thus, pediatric patients with active IBD exhibited increased pro-inflammatory and decreased pro-stabilizing CS/DS isomer composition, and future studies are needed to determine whether changes in the CS/DS-GAG composition play a pathogenic role in IBD.

## Introduction

Inflammatory bowel disease (IBD) is a chronic inflammatory condition of the gastrointestinal (GI) tract that affects more than 7 million people worldwide^1,2^. IBD is generally classified into two main subtypes, Crohn’s disease (CD) and ulcerative colitis (UC), with a smaller, third subset encompassing individuals with IBD-unclassified. Despite the increasing emergence of new therapeutic options, including state-of-the-art biologic therapies and small molecule inhibitors that target specific components of the immune response^3,4^, IBD continues to prove difficult to control, with many patients requiring multiple immunosuppressive medications and surgery^5–7^. Mucosal healing has emerged as a key therapeutic goal in the clinical management of IBD^3,8^, as it is associated with sustained clinical remission (CR), reduced hospitalizations, resection-free survival, and decreased cancer rates^9–11^. However, our ability to achieve this outcome for most patients remains limited by our incomplete understanding of underlying mechanisms driving this disease.

Increasing evidence suggests that the pathogenesis of IBD involves a combination of genetic predisposition, environmental triggers, immune dysregulation, and altered intestinal barrier integrity^12–14^. In light of recent work suggesting that intestinal barrier dysfunction plays an important role in IBD pathogenesis^15–17^, strategies aimed at restoring and maintaining intestinal barrier function may represent a novel means to improve disease management and help achieve mucosal healing. Crucial to the integrity of the intestinal barrier is the intestinal extracellular matrix (ECM), a complex, organized network of proteins and glycans that provides physical protection and scaffolding to this tissue^18–20^. The ECM dynamically regulates the ability of the intestinal barrier to interact with the surrounding environment and gut microbiome, and is fundamental in cell-to-cell communication via its close interactions with integrins and related molecules^21–23^. Specific ECM scaffolding components, including proteoglycans (PGs) and their attached glycosaminoglycan (GAG) chains, can alter the intestinal microbiota^24^, suppress the expression of pro-inflammatory cytokines^25,26^, and participate in other mucosal inflammatory processes that are pathologic hallmarks of IBD^18,19,27^. Based on these considerations, the ECM has emerged as a potential therapeutic target to promote mucosal healing in IBD.

**Figure 1:**
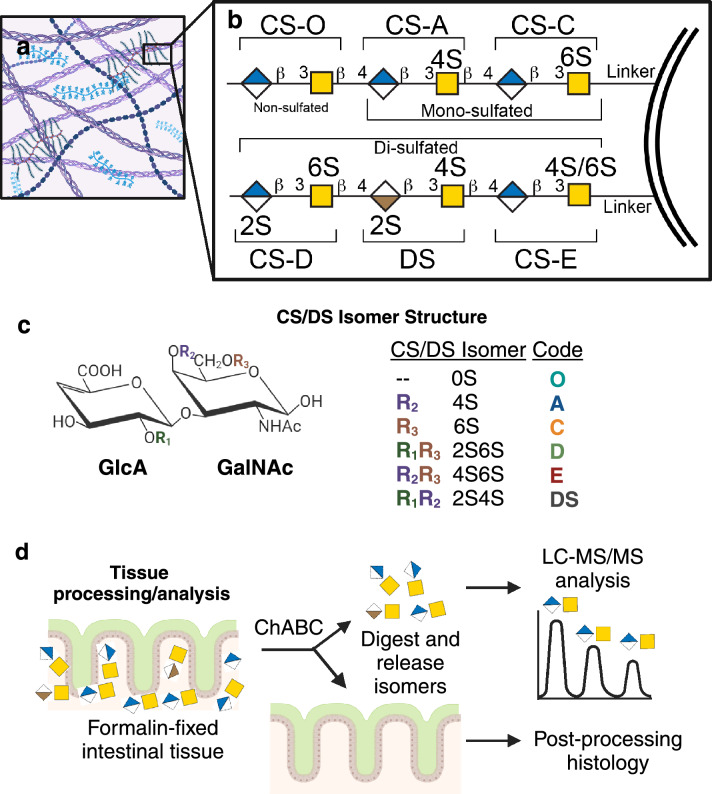
Human intestinal extracellular matrix contains chondroitin sulfate (CS) and dermatan sulfate (DS) glycosaminoglycan isomers. Diagram of the (**a**) extracellular matrix highlighting (**b**) intestinal CS proteoglycans and their attached CS and DS glycosaminoglycan (CS/DS-GAG) chains, including (**c**) non-sulfated [CS-O (0S)], mono-sulfated [CS-C (6S) and CS-A (4S)], and di-sulfated [DS (2S4S), CS-E (4S6S), and CS-D (2S6S)] isomer units. (**d**) Outline of tissue processing in which formalin-fixed and paraffin embedded (FFPE) intestinal biopsies taken during endoscopy are incubated with ChondroitinaseABC (ChABC) enzyme to digest all CS/DS-GAG chains into their individual isomer subunits, which are then collected, purified, and quantitatively analyzed by liquid chromatography-tandem mass spectrometry (LC–MS/MS) (Fig. S1). Post-processing histology is then performed on digested tissues.

Chondroitin sulfate proteoglycans (CSPGs), with their core proteins and attached chondroitin and dermatan sulfate-glycosaminoglycan (CS/DS-GAG) chains, are abundant components of the intestinal ECM (Fig. 1a). CS/DS-GAGs consist of repeating, variably sulfated disaccharides^28^, each of which are identified by unique sulfation modifications that associate with biological functions specific to the CS/DS isomer (Fig. 1b,c). The mono-sulfated CS-A (4S) isomer, which has a sulfate on the 4th carbon (4S) of the *N*-acetylgalactosamine (GalNAc), is proposed to contribute to matrix structural stability^29–31^. The di-sulfated CS-B (2S4S) isomer, also commonly identified as DS, is sulfated on the 2nd carbon of the Iduronic acid (IdoA) and 4th carbon of the GalNAc (2S4S) and is implicated in matrix assembly, wound healing, and tissue regeneration^32–34^. This tendency of structurally distinct sugars to share functionality extends to the mono-sulfated CS-C, 6th carbon sulfate attachment to GalNAc (6S), and the di-sulfated CS-E, 4th and 6th sulfate attachment to the GalNAc (4S6S), both of which are associated with inflammatory responses and accelerated cell turnover^35–40^, and the CS-C (6S) isomer is uniquely involved in matrix plasticity and loss of tissue rigidity^29–31^. The di-sulfated CS-D (2S6S) isomer is involved in neuroregeneration^41^, and finally, the non-sulfated CS-O (0S) isomer is suggested to play a key role in regulating tissue diffusion and permeability of charged molecules within the extracellular space of peripheral and central tissues^42,43^. The relative abundance of each CS/DS isomer incorporated into the CS/DS-GAG chain influences the interaction between different cell types and their surrounding environment, and moreover, appears altered in multiple inflammatory disease states^35,44–47^.

In addition to the relative abundance of these six CS/DS isomers, the average number of sulfate attachments per disaccharide unit (i.e., net sulfation) of CS/DS-GAGs is a useful index of ECM physicochemical properties. For example, the brain of patients with Alzheimer’s disease (AD) was recently reported to exhibit ‘hypersulfation’ of CS/DS-GAGs in the middle frontal gyrus in associated with AD neuropathology accumulation^35^, suggesting CS/DS-GAGs may influence both the binding and diffusion of extracellular factors within the demented brain. In this study, we explored the possibility that individuals with IBD may exhibit similar changes in intestinal CS/DS-GAGs, as observed in other inflammatory conditions, shifting the ECM to a hypersulfated, pro-inflammatory CS/DS-GAG isomer composition in involved intestines. We first performed a detailed analysis of CS/DS-GAG sulfation pattern in the human intestine using mass spectrometry methodology^35,54^ to analyze duodenum, ileum, and colon CS/DS-GAG sulfation patterns in healthy pediatric and adult patients without intestinal inflammation (Fig. 1d). Based on observations that IBD associates with decreased intestinal barrier functioning and corresponding immune cell infiltration^48,49^, combined with findings that pathologic changes in the intestinal ECM can compromise mucous barrier function, interfere with glycan-lectin interactions, and alter mucosal immunity and gut microbiota^50–53^, we then asked whether IBD associates with alterations in CS/DS-GAG sulfation patterning (and thus biological function) of intestinal ECM matrices. We accomplished this by comparing the CS/DS-GAG sulfation code in healthy patients to that of cohorts of pediatric patients with active, untreated colonic IBD and with colonic IBD in CR. Our primary goal was to first determine whether areas of inflamed colon exhibit increased pro-inflammatory CS/DS isomers and decreased pro-stabilizing CS/DS isomers, and then explore the role of pro-inflammatory changes in the composition of the colonic ECM in IBD pathogenesis.

## Results

### Region-specific intestinal CS/DS-GAG composition in pediatric patients without intestinal inflammation

**Figure 2:**
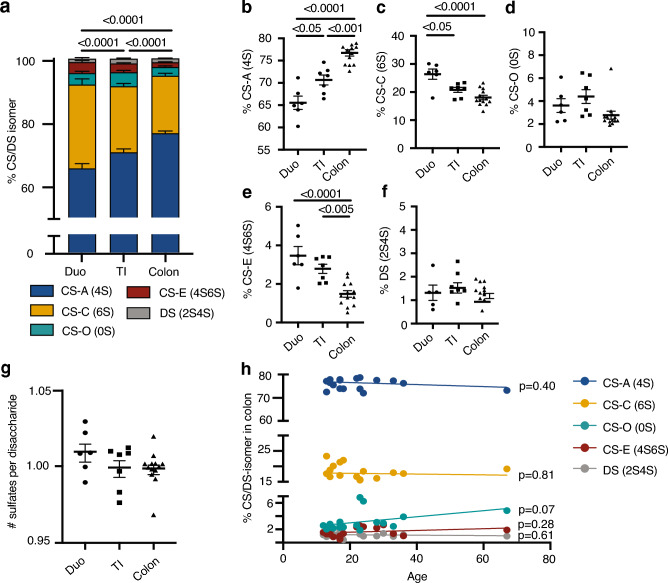
Intestinal CS/DS-GAG sulfation patterns in normal human bowel. (**a**) CS/DS isomer abundance in 13 pediatric patients without intestinal inflammation in intestinal biopsies from duodenum (duo, n = 6), terminal ileum (TI, n = 7), and colon tissue (n = 13) (p-values represent difference in the overall isomer composition). Isomer percentage compared in each area of bowel (duo, TI, and colon) for (**b**) CS-A (4S), (**c**) CS-C (6S), (**d**) CS-O (0S), (**e**) CS-E (4S6S), and (**f**) DS (2S4S) (Table S1). (**g**) Net sulfation patterns (i.e., average number of sulfate groups per individual disaccharide unit) in each location of bowel in pediatric patients (Table S1). (**h**) Colonic CS/DS isomer abundance in the above 13 pediatric patients and 6 adult patients without intestinal inflammation plotted by age. p-values not listed in (**b**–**g**) are > 0.05. Error bars represent SEM.

The relative abundance of each of the six intestinal CS/DS isomers (Fig. 1a–c), which comprises the intestinal CS/DS-GAG ‘sulfation code,’ was determined by mass spectrometry (Fig. S1, Fig. 1d) from 13 healthy pediatric/young adult patients without intestinal inflammation (Table 1, age range 12–23) in the duodenum (duo) (n = 6), terminal ileum (TI) (n = 7), and left colon (n = 13) (Fig. 2a). Consistent with what is observed in other tissues (e.g., brain^35^), the abundance of CS-A (4S) was consistently higher than any other isomer throughout the gastrointestinal tract (Fig. 2a), with the relative abundance of CS-A (4S) increasing from proximal to distal intestine (Fig. 2b, Table S1). CS-C (6S) was the second most abundant isomer found throughout the intestines and exhibited a decreased expression in the colon compared to the duodenum and ileum of the small intestine (Fig. 2c, Table S1). CS-O (0S), CS-E (4S6S), and DS (2S4S) comprised < 10% of total CS isomers throughout the intestine (Fig. 2d–f, Table S1), while CS-D (2S6S) was above the level of detection but below the level of quantification, and thus was not included in our analysis (Fig. S1). We did not observe region-dependent differences in CS/DS-sulfation, with the average number of sulfate groups per CS isomer remaining near an average of 1.0 sulfate per CS/DS isomer irrespective of intestinal location (Fig. 2g, Table S1). Collectively, these results show region-dependent CS/DS-GAG sulfation patterning, but not net sulfation, throughout the extent of human intestines.

**Table 1:** Patient demographics.

	Control		Pediatric colonic IBD at diagnosis			Pediatric colonic IBD in clinical remission		
	Pediatric	Adult	Total	Ulcerative colitis	Crohn’s disease	Total	Ulcerative colitis	Crohn’s disease
No. of patients	13	6	32	19	13	24	13	11
Age at scope, years (mean, range)	16.7 (12–23)	36.3 (24–67)	12.8 (6–18)	13.4 (6–18)	12.0 (7–15)	13.2 (7–18)	13.1 (7–18)	13.3 (10–17)
Sex (% female)	46.2%	50.0%	28.1%	31.6%	23.1%	29.2%	23.1%	36.4%
Disease phenotype				Pancolitis: 89.4%	L3B1: 84.6% L3B1 + p: 7.7% L3B2: 7.7%		Pancolitis 84.6%	L3B1: 54.5% L3B2: 18.2% L2B1: 27.3%

Intestinal biopsy samples from 13 pediatric control patients, 6 adult control patients, 32 pediatric patients with colonic IBD at diagnosis (ulcerative colitis and Crohn’s disease with colonic involvement), and 24 pediatric patients with colonic IBD at follow up (ulcerative colitis and Crohn’s disease with colonic involvement) in clinical remission were included in the study. Montreal classification was used to categorize CD phenotype. Ulcerative colitis group in clinical remission includes one patient with indeterminant colitis. *L3B1* ileocolonic non-stricturing, non-penetrating disease. *L3B1 + p* ileocolonic non-stricturing, non-penetrating disease with perianal disease. *L3B2* ileocolonic stricturing disease. *L2B1* colonic non-stricturing, non-penetrating disease.

We next sought to determine the localization of our CS/DS-GAG matrices in human intestines. Histological staining of CS/DS-GAGs is often performed using *Wisteria floribunda* agglutinin (WFA) lectin labeling in the brain^55,56^. Although this marker shows specificity for brain CS/DS-GAGs matrices^54^, WFA cross-labels mucin-type-O glycans in peripheral tissues^57^ and therefore could not be used as a selective marker for CS/DS-GAGs in our intestinal samples. Instead, we employed an enzymatic approach to identify the location of CS/DS-GAGs in our human samples utilizing ChondroitinaseABC (ChABC). This bacterial endo- and exo-lyase is capable of digesting CS/DS-GAGs into their disaccharide units and produces an unsaturated double bond on the GlcA monomer that is not produced endogenously within the mammalian system^58,59^, and antibodies raised against this ‘neo-epitope’ are highly specific for the localization of CS/DS-GAGs^60^. To localize CS/DS-GAGs in the intestine, we stained for these unique CS/DS neoepitopes post-ChABC digestion and found that CS/DS-GAGs are primarily located in the lamina propria of the colon (Fig. S2). Additional histological identification of the CSPG biglycan demonstrates colocalization with CS/DS ‘stub’ cleavage epitopes, further confirming the presence of a CSPG-rich ECM within the human intestinal tract (Fig. S2).

### Age does not influence CS/DS-GAG sulfation patterns in patients without intestinal inflammation

Of the 13 ‘control’ patients without any gross or histologic abnormalities on endoscopic evaluation, 4 of these patients were truly healthy controls (HC) (aged 18–23) who were paid to undergo endoscopy without any GI symptoms present, while 9 were patients who had nonspecific GI symptoms that prompted endoscopic evaluation (e.g., abdominal pain) and were termed functional GI disease (FGID). Here, we report that there were no significant differences in the colon CS/DS-GAG isomer profiles between HC and FGID individuals (Fig. S3, Table S2), suggesting that GI symptoms that associate with normal intestinal histopathology are not driven by changes in intestinal CS/DS-GAG patterning. Given the lack of a difference in colon CS/DS-GAGs, both HC and FGID patients are included as controls when comparing to patients with IBD.

In the brain, CS/DS-GAG sulfation patterns are significantly influenced by age^31,54^. To investigate whether human intestinal CS/DS-GAG composition is also affected by age, we compared isomer patterns from the colons of adult patients (Table 1, n = 6, age range 24–67) to those of pediatric patients without IBD described above. This analysis showed no significant correlation between patient age and the abundance of any individual CS/DS isomer (Fig. 2h). These findings suggest that after 12 years of age, the colon exhibits a similar CS/DS-GAG matrix composition throughout adulthood in individuals without intestinal inflammation, strengthening the idea that changes in the matrix composition may induce or be associated with inflammation.

### Patients with active IBD demonstrate altered colonic CS/DS-GAG composition

**Figure 3:**
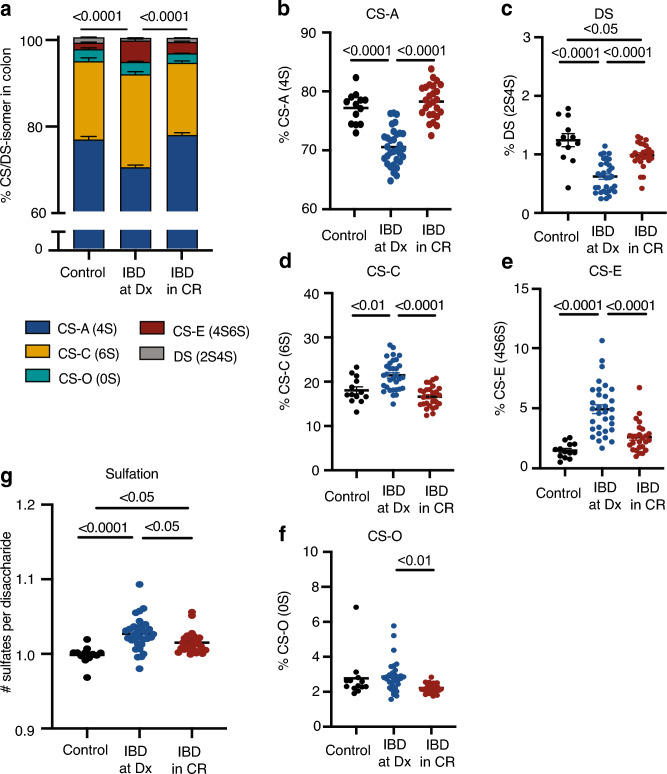
Colonic CS/DS-GAG isomer profiles are altered in active IBD. (**a**) The relative abundance of CS/DS isomers in the colons of the same control patients in Fig. 1a (n = 13) compared to individuals with IBD with colonic involvement (UC and CD colitis) at diagnosis with active clinical and histopathologic disease (IBD at Dx, n = 32), and individuals with IBD with colonic involvement at diagnosis who were in clinical remission on IBD therapy when biopsies were acquired (IBD in CR, n = 24) (Tables 1, 2). The relative abundance of (**b**) CS-A (4S) and (**c**) DS (2S4S) are significantly lower in patients with active colonic IBD at Dx compared to control patients, and CS-A (4S) normalizes in patients with IBD in CR compared to IBD at Dx. In contrast, although DS is increased in IBD in CR compared to IBC at DX, it remains lower than controls. Meanwhile, the relative abundance of (**d**) CS-C (6S) and (**e**) CS-E (4S6S) are significantly higher in patients with active colonic IBD at Dx compared to control patients, and both normalize in patients with IBD in CR compared to IBD at Dx. (**f**) CS-O (0S) isomer abundance is not significantly altered by active IBD compared to controls but is decreased in patients in IBD in CR. (**g**) Net sulfation per CS/DS isomer increases in IBD at Dx compared to controls patients, reduces significantly in IBD in CR compared to IBD at Dx, but remains elevated compared to controls. p-values not listed in (**b**–**g**) are > 0.05. Error bars represent SEM.

Next, we compared mass spectrometry analysis of CS/DS isomer composition of colonic biopsy samples obtained from a cohort of pediatric patients with active IBD with colonic involvement at diagnosis (IBD at Dx, UC and CD colitis/ileocolonic disease, n = 32) to values obtained from healthy pediatric controls (n = 13) (Fig. 3a, Table 1). These patients had both active clinical IBD symptoms (defined as Pediatric Ulcerative Colitis Activity Index (PUCAI) or Pediatric Crohn’s Disease Activity Index (PCDAI) scores > 10) and endoscopic and histologic evidence of chronic and/or active inflammation on biopsies from the left colon. Most patients with UC had pancolitis (89.4%), while most patients with CD had non-penetrating, non-stricturing ileocolonic disease without perianal disease based on the Montreal classification (84.6%) (Table 1). Compared to healthy control patients, patients with IBD at Dx were significantly younger and had a more male predominance (Table 1).

In pediatric patients with IBD at Dx, we found a significant reduction in the abundance of CS-A (4S) (Fig. 3b, Table 2) and DS (2S4S) isomers (Fig. 3c, Table 2). In contrast, CS-C (6S) (Fig. 3d, Table 2) and CS-E (4S6S) (Fig. 3e, Table 2) isomers were increased. Although the relative abundance of sulfated CS/DS isomers were shifted in the IBD at Dx population when compared to controls (Fig. 3b–e), the non-sulfated CS-O (0S) isomer was unaffected by active IBD (Fig. 3f, Table 2). Interestingly, the colonic CS-GAG composition in patients with UC compared to CD with colonic involvement was not significantly different at diagnosis (Fig. S4, Table S3), implying common CS/DS-GAG-related mechanisms underlying both IBD disorders.

**Table 2:** Composition of CS/DS isomers in the colon.

% CS/DS isomers (mean ± SEM)	Controls	IBD at Dx	IBD in CR	p-value		
				Control vs IBD at Dx	Control vs IBD in CR	IBD at Dx vs. IBD in CR
CS-A (4S)	76.5 ± 0.7	70.1 ± 0.5	77.6 ± 0.5	p < 0.0001	p = 0.51	p < 0.0001
CS-C (6S)	18.1 ± 0.8	21.5 ± 0.6	16.6 ± 0.5	p = 0.0027	p = 0.33	p < 0.0001
CS-O (0S)	2.7 ± 0.4	2.9 ± 0.2	2.2 ± 0.1	p = 0.94	p = 0.12	p = 0.0099
CS-E (4S6S)	1.5 ± 0.2	4.9 ± 0.4	2.6 ± 0.2	p < 0.0001	p = 0.12	p < 0.0001
DS (2S4S)	1.2 ± 0.11	0.6 ± 0.05	1.0 ± 0.04	p < 0.0001	p = 0.0215	p < 0.0001
Avg. # sulfates per CS/DS	0.999 ± 0.011	1.027 ± 0.021	1.015 ± 0.014	p < 0.0001	p = 0.0194	p = 0.0357

Relative percentages of colonic CS/DS isomers among control patients (Control), patients with IBD at diagnosis (IBD at Dx), and patients with IBD in clinical remission (IBD in CR).

The notable shifts in colon CS/DS-GAGs to di-sulfated CS/DS isomers in IBD patients also resulted in a striking shift in overall CS/DS-GAG net sulfation. Compared to healthy controls, patients with IBD (UC and CD with colonic involvement) exhibit hypersulfated colonic CS/DS-GAGs (Fig. 3g, Table 2), driven primarily by a marked increase in the di-sulfated pro-inflammatory CS-E (4S6S) isomer (Fig. 3e, Table 2). The aberrant changes to CS/DS-GAG sulfation patterning and hypersulfation associated with IBD at diagnosis showed a strong trend to decrease the total abundance of colon CS/DS-GAGs (Fig. S5). Overall, pediatric patients with active IBD at diagnosis with colonic involvement exhibit pathologic alterations in their CS/DS-GAG sulfation patterning that favor a more inflammatory and less stable matrix composition compared to individuals without IBD.

### CR of IBD associates with normalization of colonic CS/DS-GAG profiles

To determine whether CR of IBD (PUCAI/PCDAI < 10) also associates with changes in the aberrant CS/DS-GAG composition present in IBD at Dx, we analyzed the colonic CS/DS isomer composition in biopsies from restaging endoscopy in patients with colonic IBD while in CR (n = 24, Table 3). Patients with IBD in CR exhibit CS/DS isomer profiles similar to that observed in healthy controls (Fig. 3a). Specifically, compared to IBD patients at diagnosis, IBD patients in CR exhibited an increase in CS-A (4S) (Fig. 3b, Table 2) and DS (2S4S) (Fig. 3c, Table 2). Conversely, the abundance of CS-C (6S) (Fig. 3d, Table 2) and CS-E (4S6S) (Fig. 3e, Table 2) in IBD patients in CR decreased when compared to IBD at Dx patients with clinically active disease. Nevertheless, the CS/DS-hypersulfation characteristic of intestinal CS/DS isomers in active IBD remained detectable in patients in CR (Fig. 3g, Table 2).

**Table 3:** Therapy at follow up in patients with IBD in clinical remission.

	Pediatric colonic IBD in clinical remission		
	Total (n = 24)	Ulcerative colitis (n = 13)	Crohn’s disease (n = 11)
Mean length between diagnosis and follow up scope (months)	51.3	46.9	56.1
**Therapy at follow up**			
Biologic therapy	19/24 (79.2%)	11/13 (84.6%)	8/11 (72.7%)
Anti-TNFα	16/24 (66.7%)	8/13 (61.5%)	8/11 (72.7%)
ADA	9/24 (37.5%) Mono: 7/9 + IMM: 2/9	3/13 (23.1%) Mono: 3/3 + IMM: 0/3	6/11 (54.5%) Mono: 4/6 + IMM: 2/6
IFX	7/24 (29.2%) Mono: 6/7 + IMM: 1/7	5/13 (38.5%) Mono: 4/5 + IMM: 1/5	2/13 (15.3%) Mono: 2/2 + IMM: 0/2
VDZ	3/24 (12.5%)	3/13 (33.3%)	0
MTX	5/24 (20.8%) Mono: 1/5 + SCD: 1/5 + ADA: 2/5 + IFX: 1/5	1/13 (30.8%) + IFX: 1/1	4/11 (36.4%) Mono: 1/4 + SCD: 1/4 + ADA: 2/4
Mesal	2/24 (8.3%)	2/13 (22.2%)	0
SCD	2/24 (8.3%) Mono: 1/2 + MTX: 1/2	0	2/11 (18.2%) Mono: 1/2 + MTX: 1/2

*Anti-TNFα* anti-tumor necrosis factor α, *ADA* adalimumab, *IFX* infliximab, *Mono* monotherapy, *IMM* immunomodulator (methotrexate, 6-mercaptopurine, or azathioprine), *VDZ* vedolizumab, *Mesal* mesalamine/5-aminosalicylic acid, *MTX* methotrexate, *SCD* specific carbohydrate diet.

We also report that there were no significant age differences or sex differences between the IBD at Dx and IBD in CR groups (Table 1). The mean duration between diagnosis and time of follow up scope was 51.3 months, with no difference between UC and CD, and the majority of these patients at follow up in CR were on biologic therapies (Table 3). Notably, the colonic isomer profiles of IBD patients in CR nearly phenocopy those of control patients (Fig. 3a, Table 2), with the exception that DS (2S4S) was slightly lower in IBD patients in CR than in controls (Fig. 3c, Table 2). Furthermore, the isomer profile of these 24 patients with colonic IBD in CR were comparable to 14 patients who were in endoscopic remission (defined as absence of gross and histologic inflammation on endoscopic evaluation) (Fig. S6, Table S4).

### Pathogenic shifts in colonic CS/DS-GAG sulfation patterns correlate with histologic and clinical IBD activity

**Figure 4:**
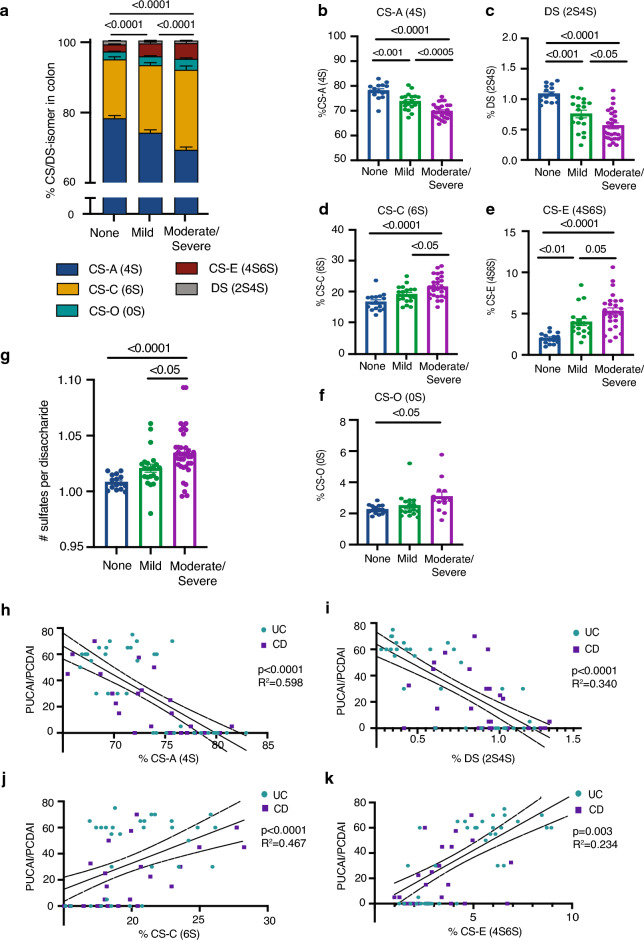
Changes in colonic CS/DS-GAG sulfation patterns correlate with histopathologic and clinical IBD activity. (**a**) The relative abundance of CS/DS isomers is significantly affected by the degree of histopathologic inflammation (defined as none, mild, or moderate/severe) in patients with IBD. The abundance of pro-stabilizing isomers (**b**) CS-A (4S) and (**c**) DS (2S4S) in colonic tissue from patients with and without IBD decreases in a stepwise fashion with the degree of pathologic inflammation, while the abundance of pro-inflammatory isomers (**d**) CS-C (6S) and (**e**) CS-E (4S6S) increases in a stepwise fashion in association with the degree of pathologic inflammation. In contrast, (**f**) CS-O (0S) abundance is not as affected by the degree of pathologic inflammation. (**g**) The degree of CS/DS sulfation increases with the degree of histopathologic inflammation. Clinical IBD activity in patients with IBD with colonic involvement (UC and CD colitis), as measured by the Pediatric Ulcerative Colitis Activity Index and the Pediatric Crohn’s Disease Activity Index (PUCAI and PCDAI) scores inversely correlates with the abundance of pro-stabilizing isomers (**h**) CS-A (4S) and (**i**) DS (2S4S) in the colon. Conversely, PUCAI/PCDAI scores increase in proportion to the abundance of pro-inflammatory isomers (**j**) CS-C (6S) and (**k**) CS-E (4S6S). p-values not listed in (**b**–**g**) are > 0.05. Error bars represent SEM.

Beyond the discovery that colonic CS/DS isomers are altered in patients with active IBD (Fig. 3, Table 2), we also found that the abundance of these isomers change significantly in proportion to the degree of histologic inflammation present in the colon (Fig. 4a, Table S5). Specifically, the levels of CS-A (4S) and DS (2S4S), which are decreased in active IBD, also decrease in proportion to the degree of histologic inflammation and appeared lower in patients with moderate/severe colonic inflammation than in patients with lesser degrees of inflammation (Fig. 4b,c). Conversely, the abundance of CS-C (6S) and CS-E (4S6S), which are higher in active IBD patients, also increased in proportion to the degree of histologic inflammation present in the colon (Fig. 4d,e). CS-O was not significantly affected by histologic inflammation between patients with mild compared to moderate/severe inflammation (Fig. 4f).

In addition to the correlation between CS/DS isomer sulfation patterns and histologic IBD activity, we also identified a significant correlation between CS/DS isomers and clinical IBD activity. Clinical IBD activity in patients with IBD with colonic disease, as defined by the PUCAI score for UC patients or the PCDAI score for CD patients, inversely correlated with the abundance of the CS-A (4S) and DS (2S4S) isomers, which are decreased in active disease (Fig. 4h,i), and positively correlated with the abundance of the CS-C (6S) and CS-E (4S6S) isomers, which are increased in active disease (Fig. 4j,k).

## Discussion

To our knowledge, our use of mass spectrometry to analyze human intestinal CS/DS-GAGs provides the first report of intestinal CS/DS-GAG composition in both healthy and diseased states. A key goal of the current work was to characterize the CS/DS-GAG sulfation code of human intestines for the first time. We found that in young individuals with endoscopically and histologically normal intestinal tissue, CS-A (4S) is the most abundant isomer, and this isomer predominantly increases as one moves from the proximal small intestine into the colon (Fig. 2). Conversely, the abundance of CS-C (6S), the second most abundant isomer, is highest in proximal small bowel and decreases moving to the terminal ileum and colon. Stated differently, the ratio of CS-A (4S) to CS-C (6S) increases as one moves distally from the proximal small bowel to the colon (Table S1). Although the functional significance of this pattern requires additional study, it may reflect a permissive role for CS-C (6S) to facilitate epithelial cell turnover, a process that is more rapid in small intestine than colon^61,62^. Furthermore, the flatter mucosal surface of the colonic epithelium (where CS-A (4S) content is highest) may benefit from a less plastic and more stable network of CS/DS isomers compared to the villous structure of the small intestine. Additional studies are warranted to investigate these possibilities.

Next, we sought to determine how the intestinal CS/DS-GAG profile is impacted in patients with clinical IBD symptoms and active IBD mucosal inflammation. Here, we report that patients with active IBD exhibit increased expression of “pro-inflammatory” isomers (CS-C (6S) and CS-E (4S6S)) and corresponding decrease in “matrix stabilizing” isomers, (CS-A (4S) and DS (2S4S)), in the colon (Fig. 3, Table 2), a pattern that also occurs in tandem with an apparent overall reduction in CS/DS-GAG abundance (Fig. S5). Pathological changes in the composition of specific CS/DS isomers and total glycan abundance have been reported in other inflammatory disease states, including AD, spinal cord injury, cardiac reperfusion injury, and traumatic brain injury^35,40,63–65^, and GAG disruption has been associated with tumor necrosis factor alpha-positive macrophages, which are implicated in IBD^66^. In colonic IBD, isomers that are associated with macrophage infiltration and glial scarring in other tissues (CS-C (6S) and CS-E (4S6S))^39,67^ are also increased in active IBD (Fig. 3). Although causality cannot be inferred from these relationships, our findings are consistent with a model whereby IBD pathogenesis involves pathological changes in ECM composition, likely associated with immune cell infiltration and activation, and future studies are warranted to test this hypothesis.

The pattern created by repeating CS/DS isomers bound to CSPG core proteins in the ECM creates a sulfation code that influences many cellular processes, the outcome of which may contribute to the pathogenesis of specific diseases^68–70^. CS/DS-GAGs appear to be most concentrated in the lamina propria of the intestine, with biglycan as the predominant associated proteoglycan (Fig. S2). Immune cell migration, activation, and cytokine production occur in the lamina propria of the intestine^71,72^, and biglycan abundance in tissues has been shown to increase in association with active immune cell infiltration and changes in pro-inflammatory CS/DS-GAG sulfation patterning^47,73^. Thus, it is likely that the CS/DS-GAG sulfation patterning changes we observe in active IBD may be linked to immune cell infiltration and activation. Alternatively, fibroblasts are also prominent in the lamina propria and are involved in ECM synthesis and remodeling^74^. Regardless of cellular source, CS/DS-GAGs associate with the binding and presentation of extracellular cytokines and chemokines^34^ to neighboring cells, and future studies investigating the relationship between changes in CS/DS-GAG sulfation patterns and activation of localized intestinal inflammation is of intense interest.

Although IBD patients initially exhibit a shift in colon CS/DS-GAG sulfation patterning, an effect linked to both pathologic severity and clinical IBD scores (Fig. 4), we observed a remarkable normalization of these CS/DS-GAG sulfation patterning in IBD patients in CR (Fig. 3). As such, shifts in CS/DS-GAG isomer profiles appear to develop only in the setting of active IBD pathology and do not represent an underlying predisposition to developing the disease. However, unlike the normalization of CS/DS-GAG patterning in IBC at CR, the colon appears to remain hypersulfated even when CR is achieved (Fig. 3). As such, the degree of colon sulfation may serve as a disease biomarker. Further studies are warranted to follow individuals longitudinally and determine whether the degree of CS/DS-sulfation is associated with a higher likelihood of disease relapse, which can guide treatment decisions and timing of repeat endoscopic evaluation.

Preclinical data from rodent studies provide further support that CS as part of the ECM plays an active role in IBD. Specifically, oral CS administration (a combination of CS isomers, predominantly CS-A (4S)), improves clinical and histologic outcomes in a dextran sodium sulfate colitis (DSS) rat model of IBD^75^. Notably, this effect was more robust and effective compared to animals receiving 5-aminosalicylic acid administration, the current first line therapy for mild to moderate UC^75^. Additionally, DSS colitis outcomes in mice were found to be improved in response to an siRNA-based strategy that altered sulfotransferase enzymatic activity, thereby reducing the abundance of pro-inflammatory CS-E (4S6S) in the colon^36^. Similar outcomes are reported in cardiac tissue, where targeted administration of the stable DS (2S4S) isomer following a cardiac ischemic event was effective in preventing reperfusion inflammation injury^65^.

One potential confounding factor in our studies is that the control population included in this study tended to be older than patients with IBD. To address this concern, we investigated whether CS/DS isomer composition varied by age in normal subjects and found no correlation between intestinal CS/DS isomer changes with age (Fig. 2). It is therefore unlikely that the differences in CS/DS isomer profiles observed between our control patients and patients with IBD were confounded by an effect of age. This is distinct from our current knowledge about age-associated CS/DS-GAG sulfation pattern changes in the brain, which are shown to change throughout the lifespan of mice as the brain matrix “stiffens” with age and becomes less plastic^30,54^. Further studies are needed to investigate the intestinal CS/DS-GAG profiles in younger individuals to determine the “critical period” for ECM maturation in the intestines.

A second potential confounder in our study is that patients with IBD (both UC and CD) had a higher male predominance compared to the control group, which had an equal sex distribution (Table 1). While pediatric IBD (CD in particular) has been shown to have a male predominance^76^, this skew was notably pronounced in our study. Finally, we acknowledge the time from diagnosis to follow-up scope among patients with IBD in CR was variable, thus producing a third limitation in our study. Overall, there was a mean length of 51.3 months between time of diagnosis and time of biopsy acquisition for tissues that were included in this study. Because this analysis utilized banked intestinal biopsy tissues, there was no way to control for therapy duration. At our institution, patients typically have their initial restaging endoscopic evaluation performed within 6–15 months of diagnosis and continue to have restaging endoscopies every few years while they remain in CR, which explains the variable and lengthy duration of time between diagnosis and follow up biopsies in our study.

In summary, our mass spectrometry analysis decodes the CS/DS-GAG sulfation signature of the human intestine for the first time and reveals pathogenic changes of CS/DS-GAG composition in areas of inflamed bowel in IBD, with pro-inflammatory isomers displacing pro-stabilizing isomers. As these ECM changes are both strongly associated with disease activity and reversed by effective IBD treatment, priorities for future studies are to determine (1) the extent to which reversal of pathogenic intestinal ECM compositional changes can improve IBD outcomes without immunosuppression, and (2) whether the intestinal CS/DS-GAG sulfation signature can be monitored as a biomarker of IBD activity.

## Methods

### Patient selection

Intestinal biopsy samples from pediatric and young adult patients with and without IBD were obtained from deidentified, banked samples through the PREDICT, ISTAT, and LAND-HO studies (PREDICT IRB STUDY00001017, ISTAT IRB STUDY00001308, and LAND-HO IRB STUDY00002018). Experiments were approved by Seattle Children’s Hospital and University of Washington Institutional Review Boards. All methods were conducted in accordance with relevant guidelines and regulations. Because all tissue samples and corresponding clinical information (age and gender for all patients, and diagnosis, IBD medical therapy, and PUCAI/PCDAI scoring for patients with IBD) were de-identified, informed consent for this analysis was not necessary; however, formal written consent was obtained from the individuals who participated in each of the above studies. While this was primarily a pediatric-focused analysis, young adults between 18 and 23 were included as healthy controls in the original studies because they could give their own consent to participate as healthy endoscopy subjects. We included all control biopsy samples that were available from these studies, and for patients with IBD our inclusion criteria were: biopsies taken from patients at time of diagnosis who were confirmed to have IBD who had clinically active symptoms and were not on treatment, with gross endoscopic and histologic colonic involvement, and/or biopsies from patients with IBD who had colonic involvement (endoscopically and histologically) at diagnosis who had subsequent biopsies taken on restaging evaluation on therapy while in CR. Most of these patients at time of follow up in CR were also in biochemical remission, with normal erythrocyte sedimentation rate and C-reactive protein lab values, but this was not an inclusion criterion for this study. There were few patients who had a fecal calprotectin collected at the same time as endoscopic evaluation, so this was also not used as an inclusion criterion. A total of 69 pediatric/young adult patients were included: 13 control patients, and 56 with IBD with colonic disease who met the above inclusion criteria (31 patients with ulcerative colitis, 1 patient with indeterminant colitis, and 24 with Crohn’s disease). A limited number of patients had tissues collected at diagnosis and follow up, but most samples were obtained from patients either at diagnosis or follow up. A smaller set of colonic biopsies from 6 deidentified adult control patients without IBD obtained through the University of Washington Gastrointestinal Center for Analytical Research and Exploratory Science (GICaRes) Biorepository (IRB 34095/2161) was also included for comparison to the pediatric population. All human tissue samples used in the analysis were deidentified, and the clinical information was provided for each deidentified sample.

### Pathologic and clinical IBD assessment

Researchers had access to pathology reports from each patient at the time of tissue collection/endoscopic evaluation. The degree of histological inflammation was categorized into none, mild, moderate, or severe according to the language used in the original pathology reports. Moderate and severe inflammation groups were combined in our analysis.

Clinical IBD activity scores, specifically PUCAI^77^ and PCDAI^78^, were calculated for each patient at the time of endoscopic evaluation based on retrospective chart review. When a numeric score was not explicitly mentioned in the chart, scores were retroactively calculated using available clinical and anthropometric information. Patients were considered to have active disease if PUCAI/PCDAI score was greater than 10. PUCAI/PCDAI scores from 10 to 34 were considered mild disease, 35–64 as moderate disease, and 65 or greater as severe disease. CR of colonic IBD was defined by our study as clinical disease activity scores (PUCAI/PCDAI < 10).

### Intestinal tissue processing

Intestinal biopsy samples from the duodenum (duo), terminal ileum (TI), and left/rectosigmoid colon were formalin-fixed and paraffin embedded (FFPE), and 4 µm thick sections were cut and mounted onto slides. FFPE sections were de-waxed using xylenes (Thermo Fischer Scientific, 1330-20-07, Waltham, MA) and a series of graded ethanol washes (100%, 95%, 70%, 50%). Slides were then washed in 0.1 M phosphate buffered saline (PBS) followed by 3 × in Optima liquid chromatography/mass spectrometry (LC/MS)-grade water (Thermo Fisher Scientific, 7732-18-5, Waltham, MA) and 1 × in 50 mM ammonium bicarbonate (pH 7.6; Sigma, 09830, Burlington, MA) at room temperature. ChondroitinaseABC (ChABC) enzyme (Sigma, C3667, Burlington, MA), a combinatorial lyase/enolase that selectively degrades all CS/DS-GAG chains into individual isomers, or disaccharide units^58,59^, was reconstituted at a concentration of 500 mU/mL in 50 mM ammonium bicarbonate (pH 7.6). Using a hydrophobic pen to outline the tissue sections on each slide, reconstituted ChABC enzyme was added to cover each tissue sections (200–500 µL per slide depending on tissue surface area), and slides were incubated at 37 °C in a Thermo Fisher Scientific MaxQ4000 orbital shaker for 24 h. Afterward, the liquid incubation media was collected and spun at 14,000×*g* for 10 min at room temperature to remove any tissue debris. The supernatant was collected and then dehydrated using a Thermo Fisher Scientific SpeedVac Concentrator and the resulting product was reconstituted in 30 µL of LC/MS-grade water for liquid chromatography with tandem mass spectrometry (LC–MS/MS) analysis. Figure 1d outlines the tissue processing workflow for the intestinal biopsy samples.

### LC–MS/MS quantification of isolated CS/DS isomers

After isolating CS/DS isomers (disaccharide units) from duodenum, terminal ileum, and colon tissues as outlined above, samples were analyzed using a triple quadropole mass spectrometer equipped with electrospray ion source (Waters Xevo TQ-S, Milford, MA) operated in negative mode ionization. LC–MS/MS was performed using a Waters Acuity I-class ultraperformance liquid chromatographic system (UPLC) coupled to the Waters Xevo TQ-S system. A Hypercarb column (2.1 Å ~ 50 mm, 3 µm; Thermo Fisher Scientific, Waltham, MA) was used to resolve the isomers as described previously^54^, assigning the following multiple reaction monitoring (MRM) channels: DS (2S4S) and CS-E (4S6S), *m/z* 538 > 300; CS-A (4S), *m/z* 458 > 300; CS-O (0S) *m/z* 378 > 175; and CS-C (6S) *m/z* 458 > 282 (Fig. S1). Data was acquired and quantified using MassLynx software version 4.1 (Waters, Milford, MA). Using the MRM channels described above, the ratios between peak areas resulting from equimolar CS/DS standard mixes were normalized to the highest peak intensity and relative quantification of each isomer within a sample was achieved using a modified peak area normalization function as previously described^54^. Each sample of tissues was normalized using internal control samples that were repeated in each LC–MS/MS run. Each CS/DS isomer was expressed as a percentage of the relative abundance of each CS/DS isomer within an intestinal sample, which allowed us to account for any variation in absolute tissue size from the biopsy samples. The average number of sulfates per CS/DS isomer was computed using the following formula:

$${Avg }\# { sulfates \, per \, CS}/{DS \, isomer }=\sum_{(i=1)}^{n}(xi+wi)/\sum_{(i=1)}^{n}wi$$

*x*_*i*_ = # sulfates (0–2).

*w*_*i*_ = percent CS/DS isomer.

### Immunohistochemical staining

Following digestion with ChABC enzyme as above, slides were subjected to immunofluorescent staining, along with undigested slides. Antigen-retrieval in 10 mM sodium citrate, pH 6.0 was performed. Sections were then incubated in 0.1 M PBS followed by 0.2% Triton X-100 in PBS, then blocked in 2% donkey serum in 0.05% Triton X-100 for 37 °C for 1 h, and finally incubated overnight with mouse anti-chondroitinase 4-sulfate antibody at 1:1000 dilution (“stub,” Millipore MAB2030, Burlington, MA, United States) and/or rabbit anti-biglycan antibody at 1:1000 dilution (Invitrogen PA5-76821, Carlsbad, CA). Sections were washed, incubated for 2 h with Alexa 555-conjugated donkey anti-mouse antibody or Alexa 647-conjugated donkey anti-rabbit and then stained with DAPI. Immunofluorescence images were captured using the Keyence BZ-X800 microscope at 20 × magnification.

### Nuclear magnetic resonance (NMR) methods to quantify total CS/DS-GAGs

Sample prep: A 20 μL aliquot containing the disaccharide was diluted in 99.9% D_2_O (Cambridge Isotope Laboratories, CIL) to a final Volume of 200 μL. The sample was dried completely under reduced pressure. Following drying the sample was reconstituted in 200 μL of 99.9% D_2_O and dried completely two additional times. After the third drying cycle, the sample was diluted in 600 μL of 99.9% D_2_O and transferred to a Norell 508UP NMR tube. The sample was promptly sealed and analyzed (Fig. S4).

### NMR experiment/processing

The ERETIC2 technique (Electronic Reference to Access in Vivo Concentration) was used to measure the concentrations of disaccharide analytes. Calibration of the instrument for ERETIC2 was carried out per the manufacturer’s recommendations (see Bruker “TopSpin ERETIC2 User Manual, Version 001”) using a benzoic acid external quantitative NMR (qNMR) standard purchased from CIL. Analysis was carried out on a Bruker Avance 300 MHz NMR Spectrometer equipped with an automatic tune and match probe via the “ERETIC 2 Quantification procedure” provided by Bruker. The 90° pulse was calibrated for each sample using the automated program “pulsecal”. The optimal number of scans (NS) and relaxation delay (D1) were determined for each sample in the usual way. Data processing was completed manually on Bruker TopSpin version 4.1.3. The concentration of each sample was determined via the ERETIC software tool in TopSpin version 4.1.3, using the aforementioned benzoic acid qNMR standard as the reference and the integration of the anomeric proton resonance from each of the disaccharides. To minimize error arising from manual processing, each spectrum was processed three times and the average of the three concentrations provided was reported with error.

### Quantification of CS/DS total abundance

Using the molar concentrations of each CS/DS isomer as determined by NMR, an equal molar solution containing all six CS/DS isomers was produced. This concentrated solution of 1 mM disaccharide mix was then diluted 1:100, 1:500, 1:1000, 1:2500, and 1:5000 and ran on the mass spectrometer instrument to produce a standard curve representing total ion counts (TIC) to the molar solution, which was then converted to ng of each CS/DS isomer. TIC produced from each intestinal CS/DS peak was then computed to ng amounts, summed, and normalized to tissue area to produce the total CS/DS-GAGs extraction from each intestinal sample.

### Statistical analysis

A mixed-effects, repeated measures two-way analysis of variance (ANOVA) with matched CS/DS isomers within each patient was used for the primary analysis comparing CS/DS isomer compositional changes between intestinal locations and between individuals without intestinal inflammation, patients with active IBD, and IBD in remission in Figs. 2a, 3a, and 4a. To compare individual isomer abundance or sulfation between each bowel location in Fig. 2b–g, a secondary analysis using Tukey’s multiple comparisons test was conducted to compare individual isomer abundance between each group and each bowel location. Data from colon biopsies for each isomer in control patients was normally distributed. To compare individual isomer abundance between controls groups and IBD groups in Fig. 3b–g, a one-way ANOVA using Tukey’s multiple comparisons test was used for normally distributed data [CS-A (4S), DS (2S4S), and CS-C (6S)]. A Kruskal–Wallis nonparametric test was used for data that was not normally distributed [CS-O (0S), CS-E (4S6S), and sulfation]. To compare the severity of histologic inflammation for each isomer in Fig. 4b–g, Tukey’s multiple comparisons test was used for normally distributed data [DS (2S4S), CS-C (6S), and CS-E (4S6S)], and a Kruskal–Wallis test was used for data that was not normally distributed [CS-A (4S)]. Normality (Gaussian distribution) was tested for using the D’Agostino-Pearson test using an alpha value of 0.05.

Whether the abundance of each isomer varied with disease activity was determined by linear regression (the 95% confidence interval bands of the best-fit line was used to compare each individual CS/DS isomer to the corresponding clinical IBD severity scores). R-squared analysis was also used to determine the extent to which variance in CS/DS isomer abundance was attributable to variation in IBD severity. GraphPad Prism 9.0 (Graph Pad Software Inc., La Jolla, CA) was used to complete statistical analyses. Error bars represent the standard error of the mean. All differences were considered statistically significant at p < 0.05. An alpha value of 0.05 was used for all analyses. Investigators were blinded to group conditions during quantitative mass spectrometry analysis.

### Supplementary Information

Supplementary Information.

## Data Availability

All data are presented within the manuscript or supplementary information files.
