# Supplementary material for: Characterizing the human intestinal chondroitin sulfate glycosaminoglycan sulfation signature in inflammatory bowel disease
Source: Sci Rep. 2024 May 23;14:11839. doi: 10.1038/s41598-024-60959-x (PMC11116513; doi:10.1038/s41598-024-60959-x)
Supplement: Supplementary file 1 — Supplementary Information. [file 41598_2024_60959_MOESM1_ESM.pdf]

Supplemental Material

Characterizing the human intestinal chondroitin sulfate glycosaminoglycan sulfation signature in inflammatory bowel disease

[1,2]Kendra L. Francis, MD, [1]Hengqi (Betty) Zheng, MD, [1]David L. Suskind, MD, [3]Taylor A. Murphree, PhD, [2]Bao Anh Phan, BS, [2]Emily Quah, BS, [2]Aarun S. Hendrickson, [3]Xisheng Zhou, BS, [1]Mason Nuding, BS, [1]Alexandra Hudson, MD, [3]Miklos Guttman, PhD, [2]Gregory J. Morton, PhD, [2]Michael W. Schwartz, MD, [2,3#]Kimberly M. Alonge, PhD, and [1,2#*]Jarrad M. Scarlett, MD, PhD

[1]Department of Pediatric Gastroenterology and Hepatology, Seattle Children's Hospital, Seattle, WA, USA

[2]University of Washington Medicine Diabetes Institute, Department of Medicine, Seattle, WA, USA.

[3]Department of Medicinal Chemistry, University of Washington, Seattle, WA, USA.

[#]equal senior author contribution

***Corresponding author**

Jarrad M. Scarlett, MD, PhD

University of Washington Medicine Diabetes Institute, Department of Medicine

750 Republican St Box 358062

Seattle, Washington, 98195, USA.

Phone: +1 (206) 897 5283

Email: jarrad.scarlett@seattlechildrens.org

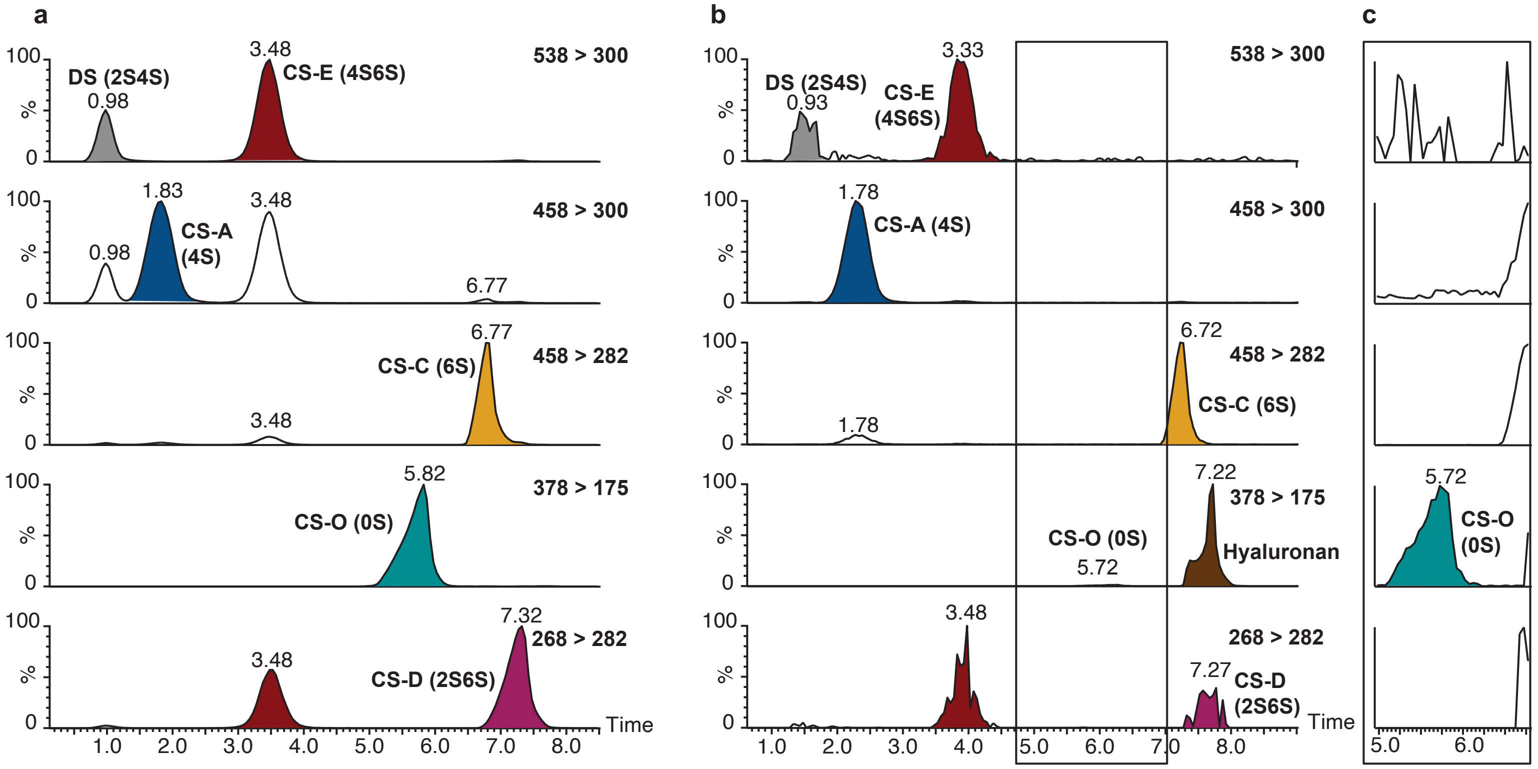

Supplemental Figure 1. Representative multiple reaction monitoring (MRM) chromatograms of the six CS/DS isomers. LC-MS/MS of (**a**) equal molar CS/DS standard mixture and (**b**) colon intestinal sample exhibit separation of each CS/DS isomer by both channel and time. ChABC exhibits Hyaluronidase (HA) activity, and the release of HA in (**b**) 378 > 175 channel indicates high enzyme activity towards CS/DS-GAGs in the intestines, while our standards do not contain HA. Insert in (**c**) shows magnification of the CS-O (0S) peak.

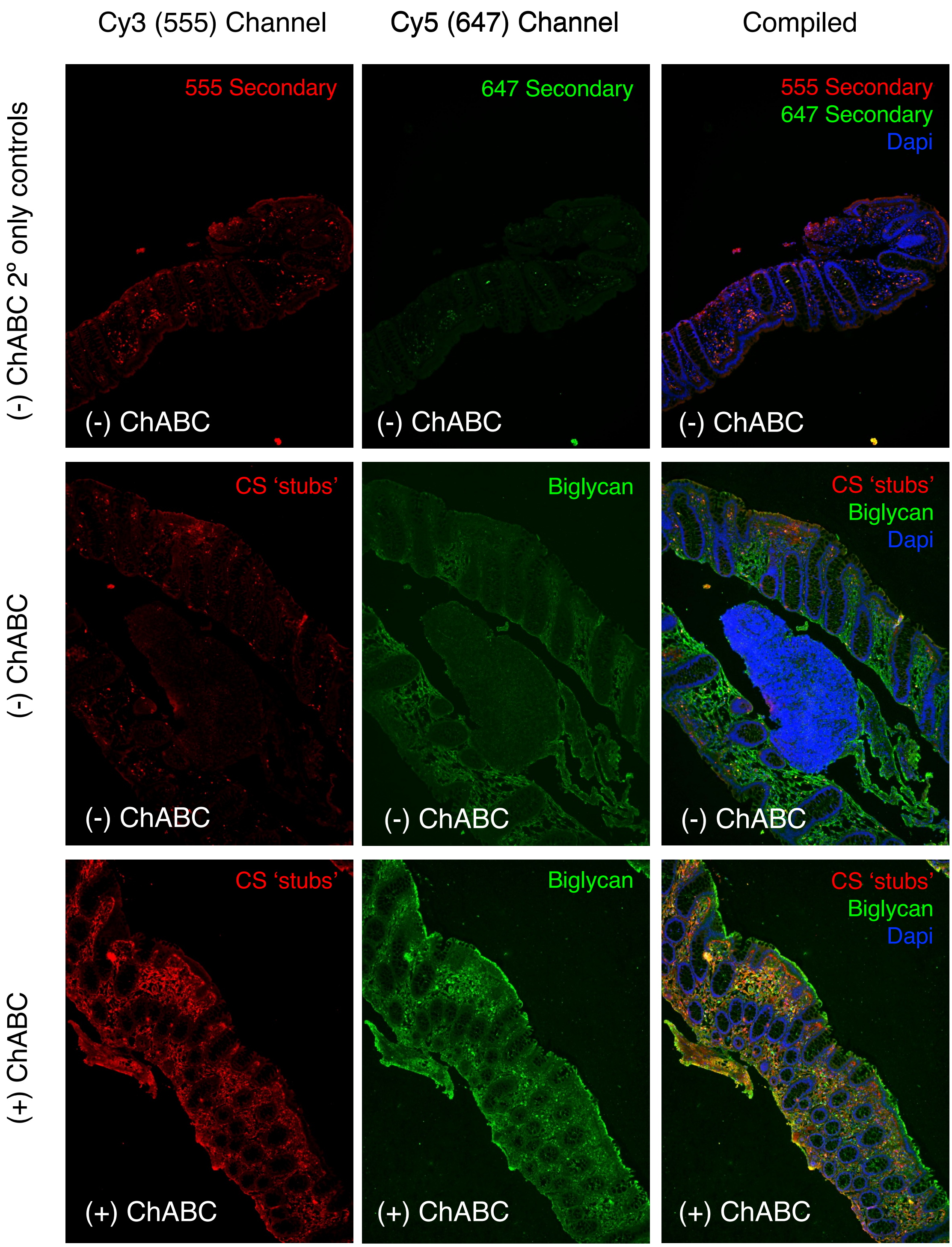

Supplemental Figure 2. Location of chondroitin sulfate (CS) in the colon and its associated proteoglycans. Staining for CS neoepitopes that are exposed only after digestion with chondroitinase ABC enzyme ("stubs") reveal that the CS/DS-GAGs are located in the lamina propria of the colon and are associated with the proteoglycan biglycan.

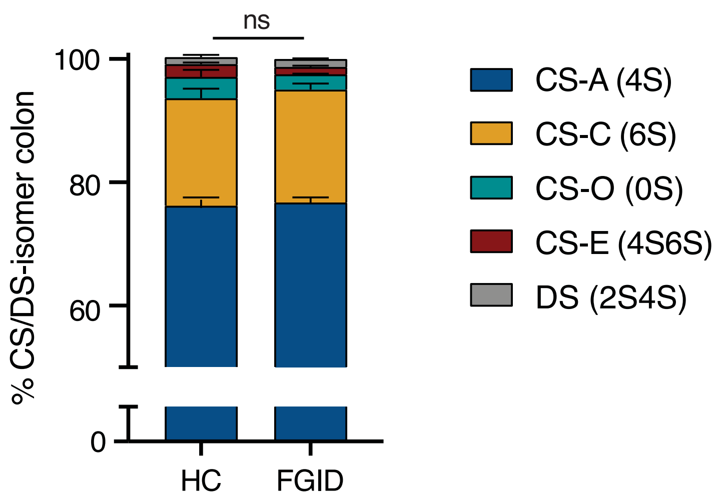

Supplemental Figure 3. Comparing the relative abundance of colonic CS/DS isomers in healthy controls (HC) and functional GI disease (FGID) patients. CS/DS colon isomer composition in healthy endoscopy controls (HC, n=4) compared to patients with functional gastrointestinal disease (FGID, n=9) who had some degree of symptomatology prompting endoscopy, but no gross or histologic changes.

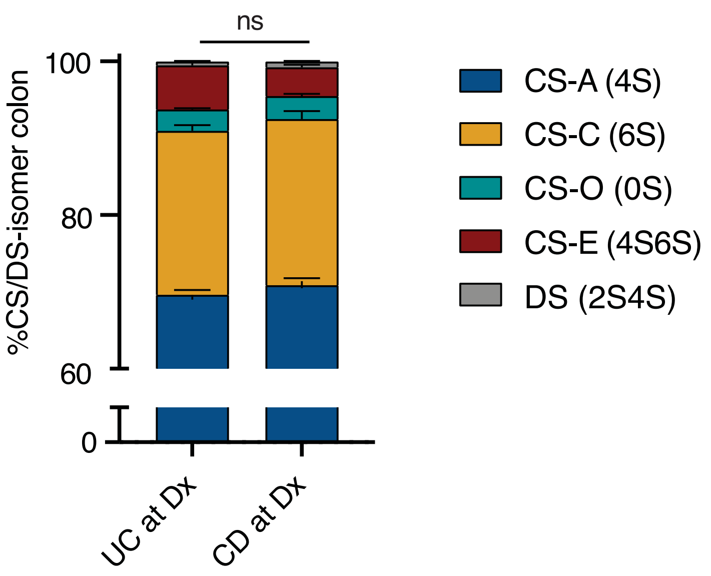

Supplemental Figure 4. Comparing the relative abundance of colonic CS/DS isomers in UC and CD at diagnosis. CS/DS colon isomer composition in patients with ulcerative colitis at diagnosis (UC at Dx, n=18) compared to patients with Crohn's disease with colonic involvement at diagnosis (CD at Dx, n=8).

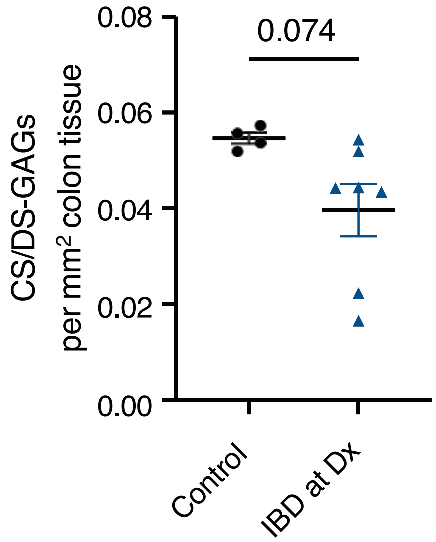

Supplemental Figure 5. Quantifying total CS/DS-GAGs isolated from the colons of controls and IBD. Total CS/DS-GAG quantification for n=4 controls and n=8 patients with IBD at diagnosis (IBD at Dx), normalized to tissue area.

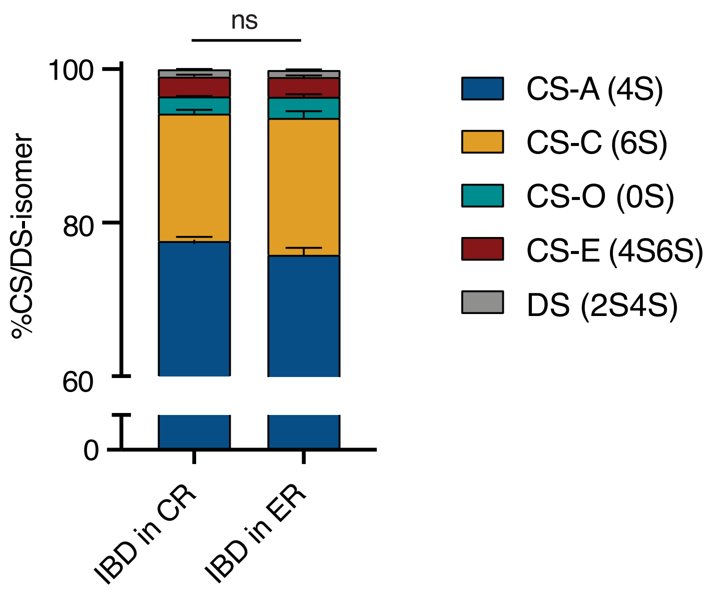

Supplemental Figure 6. Comparing the relative abundance of colonic CS/DS isomers in IBD in CR and ER. The relative abundance of colonic CS/DS isomers in patients with IBD in clinical remission (IBD in CR, n=26) at follow up compared to patients in endoscopic remission (IBD in ER, n=14) at follow up.

a

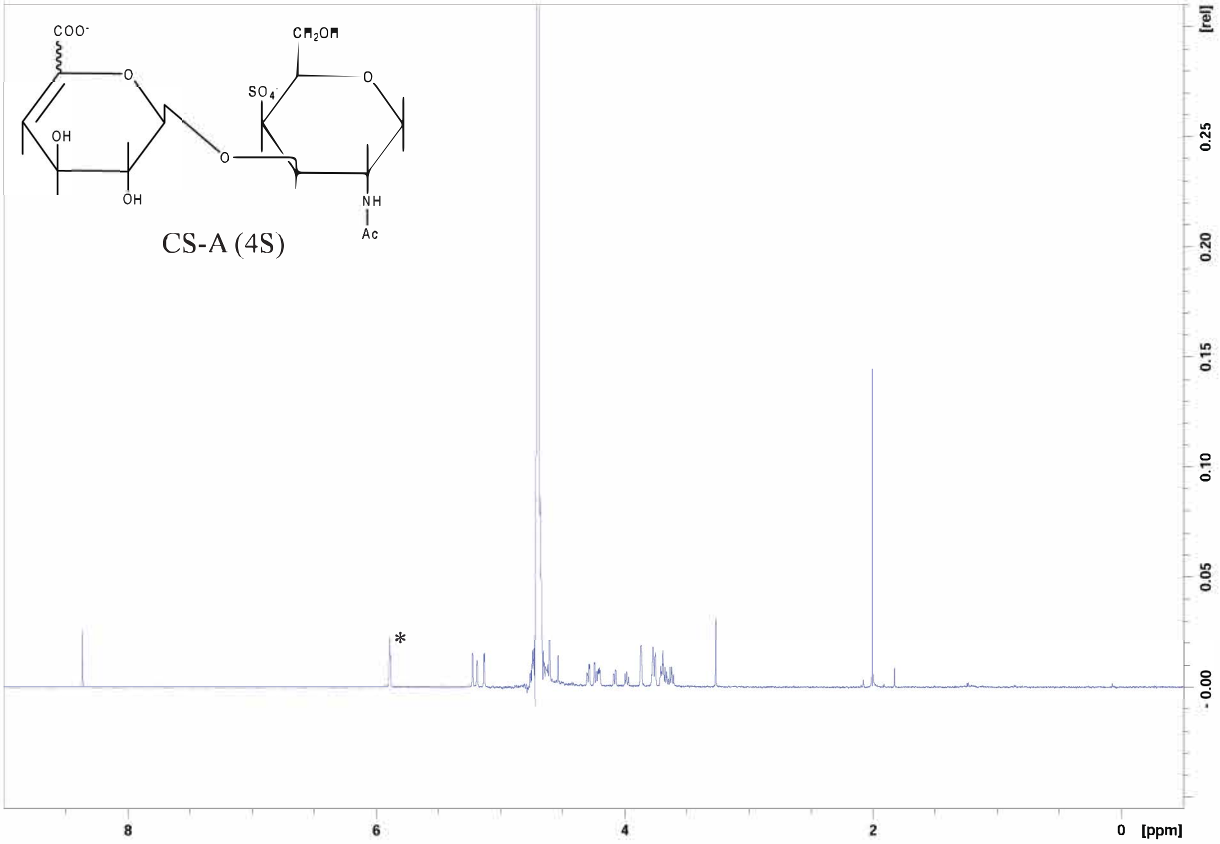

Supplemental Figure 7a-f. 1H NMR spectras of each CS/DS isomer. The spectra for H NMR spectra of (a) CS-A (4S), (b) CS-C (6S), (c) CS-O (0S), (d) CS-E (4S6S), (e) DS (2S4S), and (f) CS-D (2S6S), with each compound shown as their chemical structures shown in the insert. The integrated peak for the N-acetyl methyl peak (*) relative to a known amount of the standard (0 ppm) was used to quantify the concentration of each disaccharide.

b

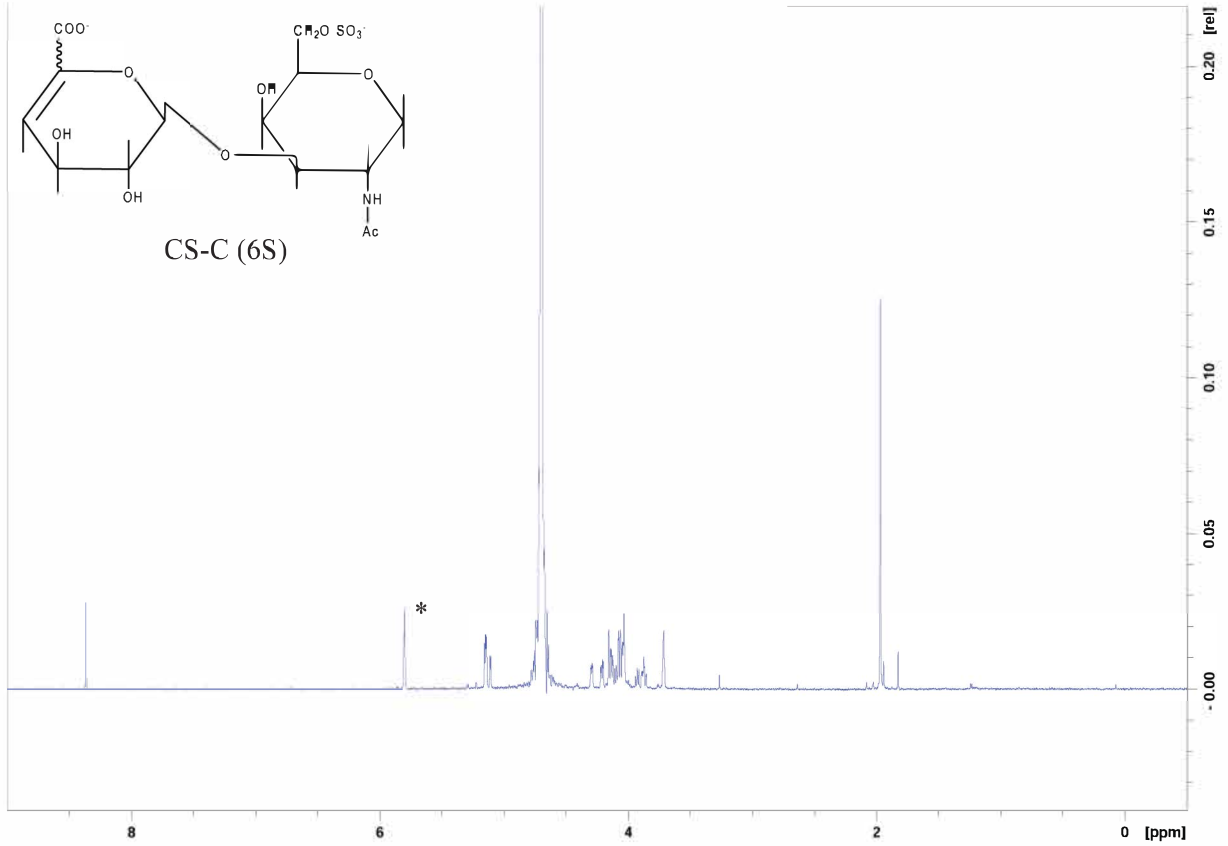
COO⁻
O
OH
OH
O
CH2O SO3⁻
OH
O
NH
Ac
CS-C (6S)
*
[rel]
0.20
0.15
0.10
0.05
0.00
8
6
4
2
0 [ppm]

c

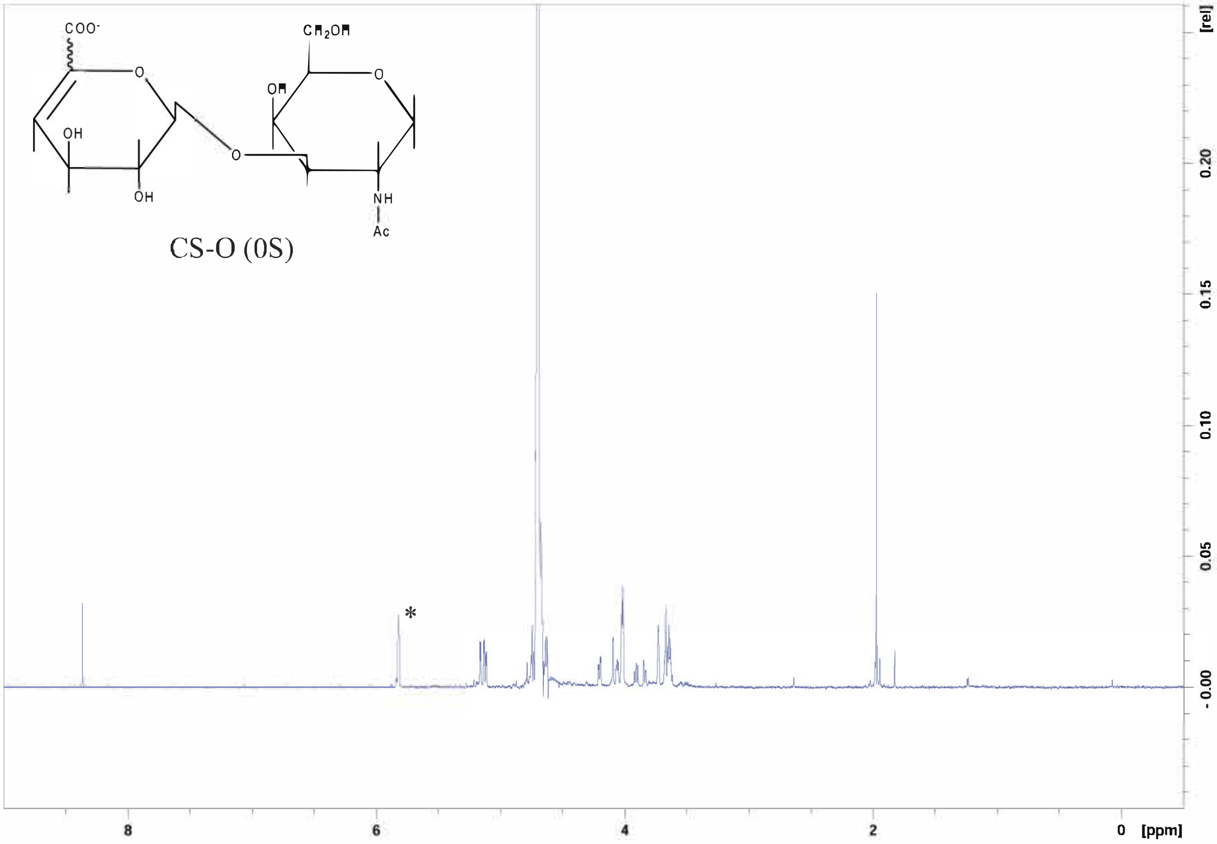

COO-
CH2OH
OH
OH
OH
O
NH
Ac
CS-O (0S)
*
[rel]
0.20
0.15
0.10
0.05
-0.00
8
6
4
2
0 [ppm]

d

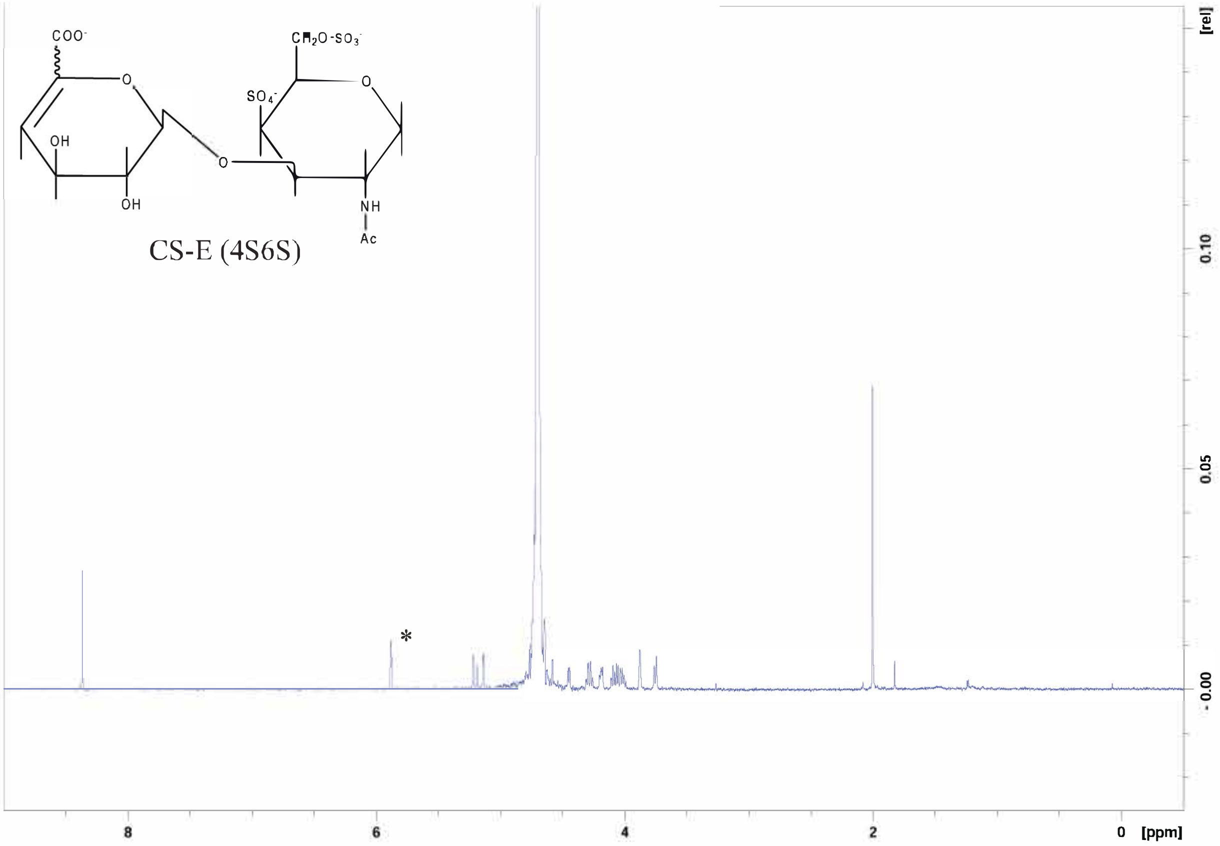

e

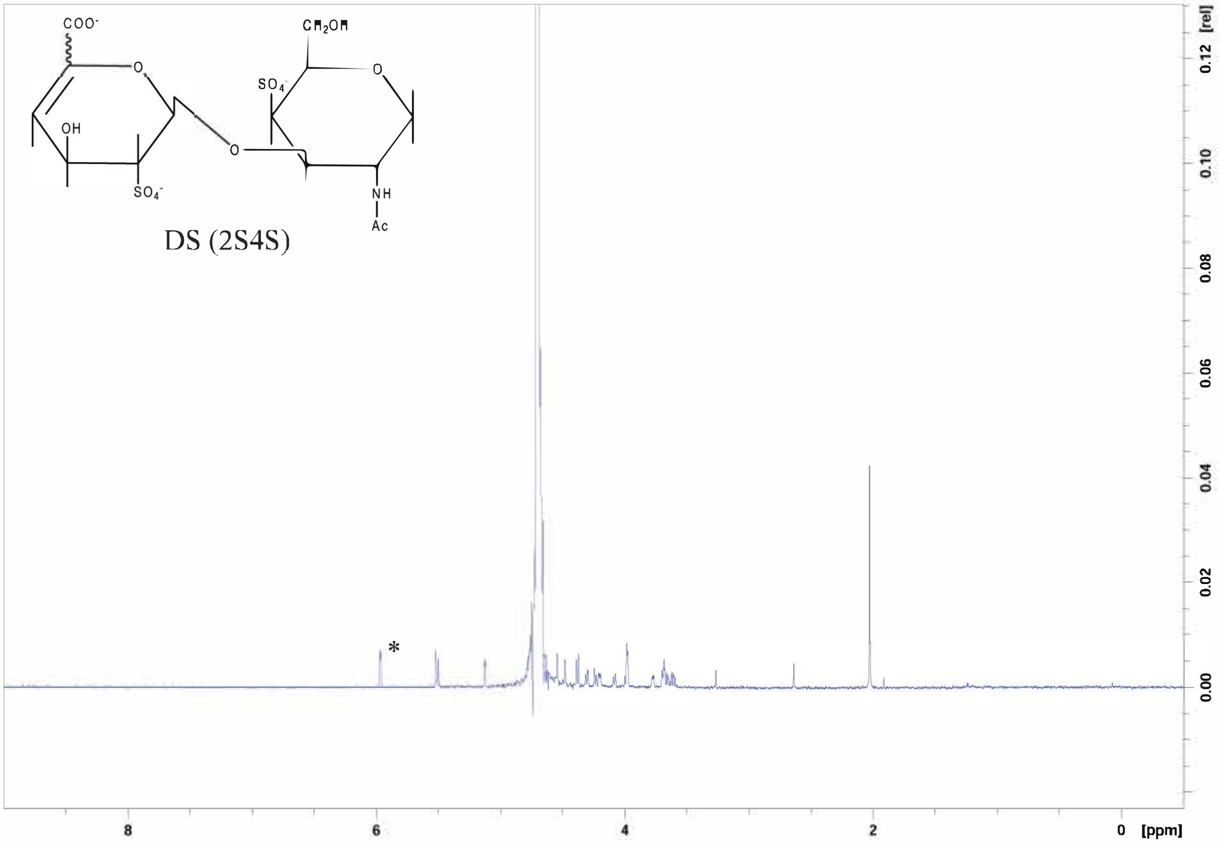

COO⁻
O
OH
SO4⁻
O
CH2OH
SO4⁻
O
NH
Ac
DS (2S4S)
*
[rel]
0.12
0.10
0.08
0.06
0.04
0.02
0.00
8
6
4
2
0 [ppm]

f

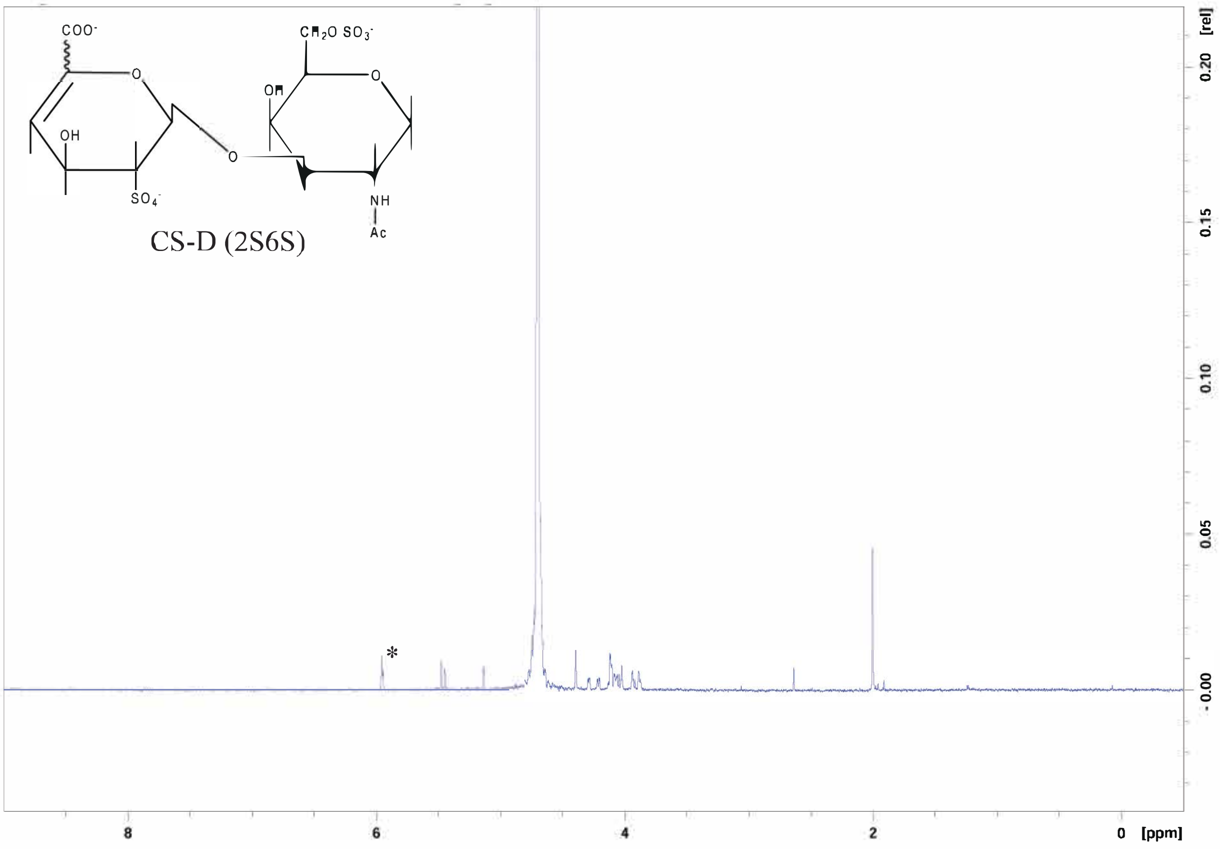

COO-
OH
SO4-
CH2O SO3-
OH
NH
Ac
CS-D (2S6S)
*
[rel]
0.20
0.15
0.10
0.05
- 0.00
8
6
4
2
0 [ppm]

**% CS/DS Isomer** mean ± SEM	**Duodenum** (Duo)	**Terminal Ileum** (TI)	**Colon**	**p-value**		
				Duo vs. TI	Duo vs. Colon	TI vs. Colon
**% CS-A (4S)**	65.4 ± 1.5	70.5 ± 1.1	76.5 ± 0.7	p=0.01	p<0.0001	p=0.0005
**% CS-C (6S)**	26.4 ± 1.8	20.8 ± 0.9	18.1 ± 0.8	p=0.0118	p<0.0001	p=0.18
**% CS-O (0S)**	3.6 ± 0.6	4.3 ± 0.6	2.7 ± 0.4	p=0.58	p=0.45	p=0.05
**% CS-E (4S6S)**	3.4 ± 0.5	2.8 ± 0.2	1.5 ± 0.2	p=0.26	p<0.0001	p=0.0041
**% DS (2S4S)**	1.1 ± 0.3	1.5 ± 0.2	1.2 ± 0.1	p=0.78	p=0.96	p=0.51
**Avg. # sulfates per CS/DS**	1.009 ± 0.006	0.991 ± 0.006	0.999 ± 0.003	p=0.33	p=0.21	p=0.99
**CS-A/CS-C ratio**	2.5 ± 0.3	3.4 ± 0.2	4.4 ± 0.2	p=0.11	p=0.0001	p=0.04

Supplemental Table 1. CS/DS isomer abundance in 13 pediatric patients without intestinal inflammation in intestinal biopsies from duodenum (duo, n=6), terminal ileum (TI, n=7), and colon tissue (n=13).

**% CS/DS Isomer** mean ± SEM	**Healthy Control**	**Functional GI Disease**	p=value
**CS-A (4S)**	76.2 ± 1.4	76.7 ± 0.9	p>0.99
**CS-C (6S)**	17.5 ± 1.5	18.3 ± 1.0	p>0.99
**CS-O (0S)**	3.4 ± 1.1	2.5 ± 0.1	p=0.96
**CS-E (4S6S)**	2.1 ± 0.3	1.2 ± 0.1	p=0.16
**DS (2S4S)**	1.1 ± 0.4	1.3 ± 0.1	p>0.99

Supplemental Table 2. The relative abundance of colonic CS/DS isomers in healthy endoscopy controls (HC, n=4) compared to patients with functional gastrointestinal disease (FGID, n=9) who had some degree of symptomatology prompting endoscopy, but no gross or histologic changes.

**% CS/DS Isomer**<br>mean ± SEM	**UC at Dx**	**CD at Dx**	p=value
**CS-A (4S)**	69.6 ± 0.6	70.8 ± 0.9	p=0.50
**CS-C (6S)**	21.3 ± 0.7	21.6 ± 1.0	p>0.99
**CS-O (0S)**	2.8 ± 0.2	2.9 ± 0.3	p>0.99
**CS-E (4S6S)**	5.7 ± 0.5	3.7 ± 0.3	p=0.07
**DS (2S4S)**	0.5 ± 0.1	0.7 ± 0.1	p>0.99

Supplemental Table 3. The relative abundance of colonic CS/DS isomers in patients with ulcerative colitis at diagnosis (UC at Dx, n=18) compared to patients with Crohn's disease with colonic involvement at diagnosis (CD at Dx, n=8).

**% CS/DS Isomer** mean ± SEM	**CR**	**ER**	p=value
**CS-A (4S)**	77.6 ± 0.5	76.0 ± 0.9	p=0.53
**CS-C (6S)**	16.6 ± 0.5	17.8 ± 0.9	p=0.28
**CS-O (0S)**	2.2 ± 0.1	2.7 ± 0.3	p=0.91
**CS-E (4S6S)**	2.6 ± 0.2	2.6 ± 0.2	p>0.99
**DS (2S4S)**	1.0 ± 0.04	0.9 ± 0.11	p>0.99

Supplemental Table 4. The relative abundance of colonic CS/DS isomers in patients with IBD at follow up in clinical remission (CR, n=26) compared to endoscopic remission (ER, n=14).

**% CS/DS Isomer** mean ± SEM	**Degree of Histologic Inflammation**			**p-value**		
	None	Mild	Moderate/ Severe	None vs. Mild	None vs. Moderate/Severe	Mild vs. Moderate/Severe
**CS-A (4S)**	78.1 ± 0.8	73.2 ± 0.8	69.7 ± 0.6	p=0.0007	p<0.0001	p=0.0004
**CS-C (6S)**	16.6 ± 0.7	19.1 ± 0.6	21.5 ± 0.7	p=0.08	p<0.0001	p=0.04
**CS-O (0S)**	2.3 ± 0.3	1.8 ± 2.5	3.1 ± 1.1	p=0.67	p=0.0221	p=0.13
**CS-E (4S6S)**	2.0 ± 0.2	4.0 ± 0.4	5.3 ± 0.4	p=0.0067	p<0.0001	p=0.05
**DS (2S4S)**	1.1 ± 0.03	0.8 ± 0.06	0.6 ± 0.05	p=0.0005	p<0.0001	p=0.0240
**Avg. # sulfates per CS/DS**	1.008 ± 0.002	1.021 ± 0.004	1.034 ± 0.004	p=0.11	p<0.0001	p=0.0211

Supplemental Table 5. The relative abundance of intestinal CS/DS isomers in 13 pediatric patients without intestinal inflammation in intestinal biopsies from duodenum (duo, n=6), terminal ileum (TI, n=7), and colon tissue (n=13).
